# Comprehensive Normative Data for Objective Vestibular Tests

**DOI:** 10.7759/cureus.40080

**Published:** 2023-06-07

**Authors:** Suman Narayana Swamy, Pradeep Yuvaraj, Nupur Pruthi, Kandavel Thennarasu, Aravind Kumar Rajasekaran

**Affiliations:** 1 Speech Pathology and Audiology, National Institute of Mental Health and Neurosciences, Bangalore, IND; 2 Neurosurgery, National Institute of Mental Health and Neurosciences, Bangalore, IND; 3 Biostatistics, National Institute of Mental Health and Neurosciences, Bangalore, IND

**Keywords:** vestibular assessment, vestibular function test, vor gain, optokinetic, smooth pursuit, saccade, caloric, vng, vhit, vemp

## Abstract

Introduction: Vestibular dysfunction is a debilitating disorder frequently encountered in neurological and otological settings. The vestibular system is a complex network between peripheral and central mechanisms. This innate complexity of the vestibular system necessitates objective test procedures for evidence-based diagnostic formulations and intervention. Objective tests aid in the evaluation of both peripheral and central vestibular pathologies. Establishing and availability of comprehensive normative data for these objective tests is crucial for clinicians and researchers alike.

Materials and methods: This is a prospective study involving 120 participants (both males and females) aged between 18 and 55 years. All participants were right-handed individuals and had no significant medical history. On pre-set protocols, cVEMP (cervical vestibular evoked myogenic potential), oVEMP (ocular vestibular evoked myogenic potential), vHIT (video head impulse test), and VNG (videonystagmography) were done.

Results: While all participants (n=120) underwent cVEMP, oVEMP, vHIT, saccade, smooth pursuit, and optokinetic tests, only 109 participants consented to the caloric test. Each test's mean, standard deviation, median, quartile, and third quartiles have been recorded. A right-left comparison yielded no significant difference on cVEMP, oVEMP, caloric test, smooth pursuit, and optokinetic test. However, few vHIT and saccade parameters did reveal significant differences.

Discussion: This study presents comprehensive normative data for cVEMP, oVEMP, vHIT, caloric test on VNG, and oculomotor tests (smooth pursuit, saccade, optokinetic) on VNG. The test results were in concordance with previously published data. The significant difference between the right and left sides in vHIT may be because of the monocular goggles used for the testing.

Conclusion: This study brings out the normative data for various vestibular tests on individuals aged between 18-55 years. This information could aid both clinicians and researchers working in the field of vestibular science.

## Introduction

Intricate peripheral structures and complex neural networks constitute the vestibular system. It gives vital input to the brain regarding where the body is, how it moves, and how it is oriented in space [1-2]. Vestibular dysfunction can be categorized into peripheral and central vestibular dysfunctions [3] and could significantly compromise the quality of life [4]. Some peripheral conditions include benign paroxysmal positional vertigo (BPPV), vestibular neuritis, and labyrinthitis. Trauma, stroke, and demyelinating conditions like multiple sclerosis are some central causes [5]. Diagnosing vestibular disorders is challenging due to factors like symptom variability and comorbidities [6-7], making objective tests necessary for an accurate and reliable diagnosis.

Objective vestibular tests evaluate the physiological integrity of the vestibular system. The commonly employed vestibular tests are cVEMP (cervical vestibular evoked myogenic potential), oVEMP (ocular vestibular evoked myogenic potential), vHIT (video head impulse test), and VNG (videonystagmography). Each test elicits responses from common and unique structures [8], providing insight into vestibular functions. Clinically, these tests aid broad lesion localization (peripheral vs central vestibular pathology), specific sites of the lesion identification (e.g. superior vs inferior vestibular nerve), and quantifying to an extant level of vestibular hypofunction. These tests also help devise treatment plans and track the disease's progression [8-10]. However, be cautioned that often supplementary information about the patient is required for better diagnostic formulation.

The normative data for various individual vestibular tests has been reported [11-14]. However, studies employed a nominal sample size [11,14] or just more than one test [12,13,15]. A comprehensive normative study including all standard vestibular tests and based on a large sample size is thus needed. This study presents comprehensive normative data for cVEMP, oVEMP, vHIT, caloric test on VNG, and oculomotor tests (smooth pursuit, saccade, optokinetic) on VNG in a single large sample.

## Materials and methods

One hundred twenty participants [both male (n=64) and female (n=56)] aged between 18 and 55 years [42.0 ± 9.73 (43.0: 35.0 - 50.0)] were recruited for the study. All participants were right-handed and reported no significant history of audio-vestibular dysfunctions. Any participants reporting neck stiffness, a history of neurological disease, alcohol dependence, and training in martial arts, yoga, dance, or gymnastics were excluded from the study. Before vestibular testing, tympanometry was performed to rule out middle ear pathology. The participants underwent cVEMP, oVEMP, vHIT, VNG (gaze, smooth pursuit, saccade, and optokinetic), and caloric tests in sequence. There was a sufficient rest period between the tests. Tests were done using well-calibrated equipment (cVEMP and oVEMP: IHS-Intelligent Hearing System Inc., Miami, Florida; vHIT: GNOtometrics-ICS Impulse USB, Denmark; VNG: ICS Chartr 200 VNG/ENG, Natus Medical Denmark). The study had been approved by the Institutional Research Board and Institutional Ethics Committee [approval number: NIMH/DO/(BS & NS DIV.)/2020-21], and written consent was sought from the participants.

Test procedure

cVEMP and oVEMP

The participants were seated on comfortable chairs. They turned their head opposite to the test ear on instruction to maintain adequate sternocleidomastoid muscle (SCM) tension when testing cVEMP [16-17]. For oVEMP, the participant was asked to fix at a point elevated approximately 30 degrees at a 60 cm distance from where they were seated [17-18]. A feedback indicator box provided biofeedback of EMG activity to the participant during the recording. The replicability of the response was determined based on two recordings. The ASCII file extracted from the best-recorded wRC-RW+LW-LCaveform was then normalized to avoid the asymmetry tonic activity of the SCM and then taken for analysis [19]. The latency (P1 and N1) and amplitude (P1-N1 for cVEMP; N1-P1 for oVEMP) are manually marked on the normalized waveform. The stimulus and recording parameters [17] for cVEMP and oVEMP are given in Table 1.

**Table 1:** Stimulus and recording parameters for cVEMP and oVEMP cVEMP: Cervical vestibular evoked myogenic potentials; oVEMP: ocular vestibular evoked myogenic potentials; Hz: Hertz; dBnHL: decibels normal hearing level; SCM: sternocleidomastoid muscle; /s: per second; EEG: electroencephalogram; EMG: electro myogram; µV: microvolts; ms: milliseconds

Stimulus and recording parameters		cVEMP	oVEMP
Type		Tone burst: 500Hz	Tone burst: 500Hz
Rate		4.1/s	4.1/s
Polarity		Rarefaction	Rarefaction
Presentation		Monoaural & ipsilateral	Monoaural & contralateral
Sweeps		100	100
Montage	Positive	Anterior 1/3 of SCM	Inferior Oblique muscle
	Negative	Sternoclavicular junction	2-3cm from the positive electrode
	Ground	Forehead	Forehead
Filter		10Hz- 1500Hz	10Hz- 1500Hz
Amplification		5000	5000
EEG window		Open window (100µV)	Open window (100µV)
EMG acceptance voltage		50 µV- 200 µV	1µV- 100 µV
Post stimulus		50ms	50ms
Pre stimulus		100ms	­100ms

vHIT

Participants were seated in a dark room; the test goggles mounted with the monocular camera on the right eye were strapped tightly to the participant's head. As instructed, the participants fixed their gaze on a point marked on the wall at a one-meter distance. The test was done in both yaw and pitch planes to stimulate all six semicircular canals (horizontal, anterior, and posterior canals on each side). The participant's head was held firmly during the test and turned quickly and unpredictably through 10-20 degrees. Impulses were delivered randomly to right and left directions until 20 impulses got accepted for each direction. Equipment software's pre-set proprietary algorithms automatically calculated average VOR gain, VOR asymmetry, and metrics for covert and overt saccades (total saccade percentage, latency, and amplitude) for all six semicircular canals. The VOR asymmetry was analysed for between the semicircular canals: right anterior-left anterior (RALA), right lateral-left lateral (RLLL), right posterior-left posterior (RPLP), left anterior-right posterior (LARP), and right anterior-left posterior (RALP).

VNG

The participants were seated in a dark room 3.8 to 4.2 feet from the light bar, and the test goggles were strapped tightly to their heads. After successful pupil detection and checking the region of interest, calibration was done (for the individual and both eyes) in both horizontal and vertical planes. The gaze, saccade, and smooth pursuit tests used 'individual eye' calibration data. The optokinetic test and caloric test used 'both eye' calibration data. 

Saccade Test

As instructed, the participants kept looking at the red light appearing on the light bar without moving their heads. The test was done on horizontal and vertical planes (10 and 15 degrees). A minimum of 20 seconds of recording was considered for each test. The equipment automatically generated average rightward and leftward peak velocity, accuracy, and latencies.

Smooth Pursuit

As instructed, the participants followed the red light on the light bar without moving their heads. Stimuli of different frequencies (20 Hz, 30 Hz, 40 Hz, 50 Hz, 60 Hz, and 70 Hz) were presented continuously, one after the other, in ascending order for both the horizontal and vertical planes. The target (red light) moved from the centre to the extreme left-to-right position on the light bar, with three cycles of displacements for each frequency. The mid-cycle of each frequency recording was selected to record right and left eye gain.

Optokinetic Test

As instructed, the participants looked at the light bar as the lights continuously crossed the field of vision at 20 degrees per second. The test was done in the right and left directions for at least 20 seconds. Nystagmus appearing near 10 seconds was a reference to calculate the average peak SPV for 10 seconds (5 seconds before and after the selected nystagmus).

Caloric Test

As instructed, the participants lay supine with their heads elevated at a 30-degree angle. In the test, the right cool, left cool, right warm, and left warm conditions were achieved by presenting a bi-thermal (24° C for cool and 50° C for warm) air irrigation to the external auditory canal. During the test, the participants kept their eyes open as much as possible for a clear recording of eye movements. Between each test (conditions), the rest period was at least five minutes. 'Jongkee's formula' was used to calculate unilateral weakness and directional preponderance. The individual right and left ear maximum SPV and total SPV for each condition too were recorded. 

UW = (RC-RW)-(LW-LC) / (RC-RW+LW-LC) × 100

DP = (RC+LW)+(RW+LC) / (RC-RW+LW-LC) × (-100)

where unilateral weakness (UW); directional preponderance (DP); right warm (RW); right cool (RC); left warm (LW); left cool (LC)

## Results

Vestibular tests were conducted on 120 participants (64 males and 56 females) with a mean age of 42±9.7 years. All 120 participants underwent cVEMP, oVEMP, vHIT, saccade, smooth pursuit, and optokinetic tests. At the same time, only 109 participants agreed to undergo a caloric test. Further in the smooth pursuit test the number of participants able to perform smooth pursuit reduced as the frequency of the stimulus increased from 20 Hz to 70 Hz (horizontal: 20 Hz- 120, 30 Hz- 113, 40Hz- 99, 50Hz- 86, 60Hz- 83, 70Hz- 77; Vertical: 20Hz- 65, 30Hz- 59, 40Hz- 45, 50Hz- 30, 60Hz- 25, 70Hz- 23).

The data were analyzed using IBM SPSS Statistics for Windows v. 28 (IBM Corp. Armonk, NY: IBM Corp). By employing descriptive statistics, the mean, standard deviation, median, and first and third quartiles are presented. The nonparametric Wilcoxon signed-rank test was used to find the right and left differences. The statistical significance was set at 0.01 for all the tests. The analysis was carried out as a whole group (both males and females) and separately for male and female participants. The descriptive statistics and the results of the comparisons between right and left for the whole group (male and female together) and individual groups (male and female separately) are given in Tables 2-8 below.

**Table 2:** cVEMP [Mean±SD (Median: Q1 – Q3)] and comparison between right and left ear in the whole group, males and females P1: Positive peak, N1: Negative peak, n: sample size, SD: standard deviation, Q1: 1st quartile, Q3: 3rd Quartile, msec: milliseconds, µV: microvolts

Ear	Right ear	Left ear	The whole group (n= 120)		Male (n= 64)		Female (n= 56)	
			Z	p-value	Z	p-value	Z	p-value
P1 Latency (msec)	16±1.2 (16:15.2-16.8)	16.09±1.31 (15.8:15.2-16.8)	-1.278	0.201	-0.156	0.876	-1.556	0.120
N1Latency (msec)	24.3±2.0 (24:23-25.8)	24.4±2.05 (24:23-25.2)	-0.925	0.355	-0.632	0.527	-0.724	0.469
Normalised P1-N1 Amplitude (µV)	23.9±8.4 (23:18.4-29.4)	23.9±8.7 (23.3:17.1-28.3)	-0.352	0.725	-0.388	0.698	-0.086	0.932

**Table 3:** oVEMP [Mean±SD (Median: Q1 – Q3)] and comparison between the right and left ear in the whole group, males and females P1: positive peak, N1: negative peak, n: sample size, SD: standard deviation, Q1: 1st quartile, Q3: 3rd Quartile, msec: milliseconds, µV: microvolts

Test measure	Right ear	Left ear	Whole group (n= 120)		Male (n= 64)		Female (n= 56)	
			Z	p-value	Z	p-value	Z	p-value
N1 Latency (msec)	12.0±0.73 (11.6:11.4-12.4)	12±0.76 (11.8:11.4-12.3)	-1.280	0.201	-0.526	0.599	-1.249	0.212
P1 Latency (msec)	16.5±1 (16.6:15.8-17.2)	16.7±1 (16.8:16.2-17.4)	-2.059	0.039	-2.401	0.016	-0.514	0.607
N1-P1 Amplitude(µV)	15.6±9 (13.4:9.1-21.2)	15.4±9.4 (12.1:8.8-20.3)	-1.006	0.314	-1.380	0.168	-0.082	0.935

**Table 4:** vHIT [Mean±SD (Median: Q1 – Q3)] and comparison between right and left ear in the whole group, males and females n: sample size, SD: standard deviation: Q1: 1st quartile, Q3: 3rd quartile, VOR: vestibular-ocular reflex gain: SCC: semi-circular canal

VOR gain	Right ear	Left ear	The whole group (n= 120)		Male (n= 64)		Female (n= 56)	
			Z	p-value	Z	p-value	Z	p-value
Anterior SCC	0.862±0.1 (0.9:0.8-0.9)	0.800±0.08 (0.8:0.7-0.9)	-6.031	<0.001	-4.293	<0.001	-4.265	<0.001
Lateral SCC	0.979±0.08 (1:0.9-1)	0.930±0.08 (0.9:0.9-1)	-7.403	<0.001	-5.235	<0.001	-5.217	<0.001
Posterior SCC	0.845±0.08 (0.8:0.8-0.9)	0.834±0.09 (0.9:0.8-0.9)	-1.415	0.157	-1.406	0.160	-0.568	0.570

**Table 5:** Caloric test [Mean±SD (Median: Q1 – Q3)] and comparison between right and left ear in the whole group, males and females n-sample size, SD-Standard deviation, Q1-1st quartile, Q3-3rd Quartile, SPV-slow phase velocity

Stimuli	Right ear	Left ear	The whole group (n= 110)		Male (n=61)		Female (n=49)	
			Z	p-value	Z	p-value	Z	p-value
Cold (SPV)	15.9±9.8 (15:8-23)	16.6±10.7 (15:8-24)	-1.255	0.210	-1.311	0.190	-0.249	0.804
Warm (SPV)	17.9±14.7 (13:7 – 26)	19.3±16.3 (16:7 – 24)	-1.385	0.166	-0.567	0.571	-1.525	0.127
Total (SPV)	33.7±22.4 (27:16-49)	35.9±24.7 (31:18 – 49)	-2.106	0.035	-1.300	0.194	-1.800	0.072

**Table 6:** Saccade peak velocity, accuracy, and latency of individual eye [Mean±SD (Median:Q1 – Q3)] and comparison between right and left eye in whole group, males, and females n-sample size, SD-Standard deviation, Q1-1st quartile, Q3-3rd Quartile, °/sec-degrees/ second, msec-milliseconds

Test	Right eye	Left eye	Whole group (n= 120)		Male (n= 64)		Female (n= 56)	
			Z	p-value	Z	p-value	Z	p-value
Horizontal 10 degrees								
Rightward peak velocity (°/sec)	391.3±53.3 (393:364.3-423.0)	406.2±57.4 (412:377.3-435.0)	-4.594	<0.001	-3.629	<0.001	-2.916	0.004
Leftward peak velocity (°/sec)	424.9±60.6 (427:391.3-460.0)	415.7±60.7 (412:379.0-450.0)	-2.427	0.015	-0.900	0.368	-2.464	0.014
Rightward accuracy	87.7±8.7 (89:82.3-93.8)	90.6±8.4 (92:86.0-96.0)	-5.131	<0.001	-3.442	<0.001	-3.825	<0 .001
Leftward accuracy	90.1±7.7 (90:85.0-95.0)	89.2±7.1 (90:85.0-94.0)	-1.710	0.087	-0.622	0.534	-1.787	0.074
Rightward Latency (msec)	172.7±35.9 (166:150.3-190.8)	170.3±34.3 (163:148.0-188.3)	-1.283	0.200	-0.130	0.896	-1.531	0.126
Leftward Latency (msec)	167.3±34 (163:148.3-181.8)	164.6±31.7 (161:145.0-180.0)	-2.310	0.021	-2.175	0.030	-1.121	0.262
Horizontal 15 degrees								
Rightward Peak velocity (°/sec)	449.7±71.4 (450:403.8-491.0)	454.6±68.2 (457:409.3-489.5)	-2.237	0.025	-1.799	0.072	-1.479	0.139
Leftward Peak velocity (°/sec)	474.5±78.6 (476:426.5-526.3)	460.6±65.9 (461:421.5-503.0)	-3.528	<0.001	-2.287	0.022	-2.623	0.009
Rightward Accuracy	88.5±7.8 (90:82.3-94.0)	90.8±7.1 (92:86.0-97.0)	-4.092	<0.001	-2.257	0.024	-3.430	<0.001
Leftward Accuracy	90.4±7.7 (91:85.0-96.0)	89.5±6.6 (90:86.0-93.0)	-2.076	0.038	-0.665	0.506	-2.190	0.028
Rightward Latency (msec)	174.8±38.7 (168:149.3-189.0)	169.8±35.8 (165:147.0-186.8)	-1.048	0.295	-0.950	0.342	-0.554	0.580
Leftward Latency (msec)	178.8±36 (174:154.0-194.5)	172.0±34.4 (166:149.3-185.8)	-4.104	<0.001	-4.129	<0.001	-1.796	0.073
Vertical 10 degrees								
Rightward Peak velocity (°/sec)	414.7±74.1 (403:365.3-460.5)	416.5±66.8 (411:379.8-446.5)	-0.375	0.707	-0.455	0.649	-0.061	0.951
Leftward Peak velocity (°/sec)	426.5±73.1 (422:377.8-474.8)	427.1±69.1 (423:382.8-466.8)	-0.169	0.866	-0.488	0.625	-0.465	0.642
Rightward Accuracy	94.3±10.0 (95:89.0-100.0)	93.8±9.0 (95:88.3-98.0)	-0.675	0.499	-0.466	0.642	-0.619	0.536
Leftward Accuracy	93.3±9.6 (95:86.3-99.0)	93.9±9.8 (95:89.3-99.0)	-0.994	0.320	-0.319	0.750	-1.130	0.258
Rightward Latency (msec)	174.8±26.8 (173:157.3-187.0)	173.8±36.3 (169:155.0-183.0	-2.978	0.003	-3.015	0.003	-1.330	0.183
Leftward Latency (msec)	183.2±31 (181:156.0-204.0)	182.5±40.2 (179:152.8-205.8)	-2.440	0.015	-2.549	0.011	-0.868	0.385
Vertical 15 degrees								
Rightward Peak velocity (°/sec)	453.5±76.1 (448:400.3-506.5)	461.3±75 (457:417.5-506.8)	-1.281	0.200	-1.491	0.136	-0.285	0.776
Leftward Peak velocity (°/sec)	464.3±74.5 (455:418.8-505.8)	468.6±68.4 (469:424.3-503.8)	-1.176	0.239	-0.204	0.838	-1.416	0.157
Rightward Accuracy	90.1±8.8 (90:85.0-97.0)	90.7±8.4 (91:85.0-96.8)	-0.472	0.637	-0.004	0.997	-0.907	0.364
Leftward Accuracy	91.1±8.8 (93:85.3-97.0)	91.9±7.7 (94:86.5-97.0)	-0.873	0.383	-0.031	0.975	-1.377	0.169
Rightward Latency (msec)	178.7±30.8 (175:160.3-192.5)	177.6±30.3 (174:160.0-189.5)	-0.653	0.513	-0.327	0.744	-0.934	0.350
Leftward Latency (msec)	183.3±34.9 (180:161.0-198.0)	182.9±32.8 (181:163.0-199.0)	-1.071	0.284	-1.105	0.269	-0.378	0.705

**Table 7:** Smooth pursuit gain for 20Hz – 70Hz [Mean±SD (Median: Q1 – Q3)] and comparison between right and left ear in the whole group, males and females n: sample size, SD: standard deviation, Q1: 1st quartile, Q3: 3rd quartile

Test parameter		Frequency					
		20Hz	30Hz	40Hz	50Hz	60Hz	70Hz
Horizontal	Right eye rightward movement	0.92±0.11 (0.9:0.85-1)	0.9±0.1 (0.9:0.84-1)	0.86±0.13 (0.85:0.77-0.97)	0.84±0.13 (0.84:0.74-0.94)	0.77±0.16 (0.78:0.68-0.88)	0.70±0.16 (0.72:0.64-0.82)
	Right eye leftward movement	0.92±0.11 (0.9:0.85-1)	0.9±0.1 (0.9:0.83-1)	0.87±0.13 (0.87:0.8-0.97)	0.85±0.13 (0.84:0.75-0.96)	0.75±0.16 (0.77:0.66-0.89)	0.70±0.17 (0.72:0.61-0.81)
	Left eye rightward movement	0.92±0.08 (0.91:0.85-1.0)	0.91±0.09 (0.91:0.85-1.0)	0.87±0.11 (0.85:0.80-0.98)	0.84±0.12 (0.85:0.75-0.95)	0.77±0.14 (0.76:0.70-0.88)	0.71±0.14 (0.72:0.65-0.81)
	Left eye leftward movement	0.91±0.08 (0.91:0.85-1.0)	0.90±0.09 (0.91:0.85-1.0)	0.86±0.11 (0.87:0.78-0.97)	0.85±0.11 (0.85:0.75-0.95)	0.76±0.15 (0.76:0.68-0.89)	0.67±0.16 (0.70:0.60-0.80)
Vertical	Right eye rightward movement	0.76±0.14 (0.77:0.65-0.87)	0.66±0.16 (0.70:0.55-0.80)	0.66±0.17 (0.68:0.50-0.81)	0.52±0.19 (0.55:0.33-0.70)	0.54±0.20 0.58:0.32-0.71)	0.51±0.20 (0.52:0.40-0.68)
	Right eye leftward movement	0.74±0.16 (0.75:0.65-0.85)	0.68±0.16 (0.67:0.56-0.80)	0.63±0.16 (0.62:0.50-0.77)	0.53±0.18 (0.53:0.39-0.72)	0.50±0.16 (0.50:0.33-0.65)	0.48±0.14 (0.54:0.35-0.60)
	Left eye rightward movement	0.77±0.13 (0.80:0.65-0.88)	0.66±0.17 (0.70:0.55-0.79)	0.66±0.17 (0.70:0.50-0.80)	0.53±0.19 (0.55:0.35-0.70)	0.53±0.20 (0.51:0.32-0.70)	0.52±0.20 (0.51:0.41-0.68)
	Left eye leftward movement	0.76±0.14 (0.80:0.68-0.85)	0.68±0.16 (0.70:0.55-0.80)	0.63±0.15 (0.61:0.50-0.75)	0.54±0.18 (0.54:0.40-0.71)	0.52±0.16 (0.55:0.37-0.65)	0.47±0.14 (0.51:0.36-0.55)

**Table 8:** Optokinetic test [Mean±SD (Median: Q1 – Q3)] and comparison between right and left ear in the whole group, males and females n: sample size, SD: standard deviation, Q1: 1st quartile, Q3: 3rd quartile, SPV: slow phase velocity

Stimuli	Right ear	Left ear	Whole group (n= 120)		Male (n= 64)		Female (n= 56)	
			Z	p-value	Z	p-value	Z	p-value
Optokinetic gain (SPV)	19.6±4.50 (20.0:17.0-22.8)	19.47±4.85 (20.0:16.0-22.0)	-0.528	0.598	-0.056	0.955	-0.987	0.324

There were no significant differences (Wilcoxon signed-rank test; significance level set at 0.01) between right- and left-side stimulation in cVEMP, oVEMP, and caloric test (Tables 2, 3, 5). However, a few parameters on vHIT, the VOR gain of the anterior and lateral canals, showed a significant difference between the right and left sides in all three groups (Table 4). On oculomotor tests, smooth pursuit and optokinetic tests showed no significant differences between the right- and left-side (Tables 7-8). In the saccade test, rightward peak velocity and rightward accuracy for the 10-degree horizontal plane, leftward peak velocity, rightward accuracy, and leftward latency or 15-degree horizontal plane showed significant differences between the right and left sides (Table 6). For additional clinical reference, the following results are reported. The amplitude asymmetry ratio (AAR) for cVEMP and oVEMP were 10.6±8 and 9.2±7.4, respectively. The mean asymmetry values for RALA, RLLL, RPLP, LARP, and RALP were 10.0±7.3, 5.9±3.9, 8.2±6, 9.0±6.4, and 8.7±5.6 respectively. The unilateral weakness (UW) and directional preponderance (DP) on the caloric test were 17.0±13.7 and 15.0±11, respectively.

## Discussion

In the current study, 120 normal participants underwent various vestibular tests. All participants had recordable cVEMP and oVEMP. Previous studies have reported an 87% to 100% response rate [20-22]. Variations in stimulus parameters could lead to different test findings in VEMP. The current study used a widely employed [20, 22-26] VEMP stimulus (500 Hz tone burst at 106 dBnHL). The P1 and N1 latencies, peak-to-peak amplitude, and amplitude asymmetry ratio observed were similar to those reported in previous studies [11, 26-27]. Amplitude normalization (for cVEMP) was performed on raw waveforms to control the differential contraction effect of right and left SCM. A raw waveform comparison could have given out erroneous values [19]. Further, there was no significant difference between right and left ear stimulation for both cVEMP and oVEMP. Previously, studies on cVEMP [20, 28-30] and oVEMP reported similar findings [18, 31].

The vHIT is a new addition to the vestibular test battery, and very few normative studies are available. Studies mainly focus on lateral semicircular canals or involve small sample sizes. The current study presents normative data for all six semicircular canals involving a large sample size. The VOR gain of the right lateral and right anterior SCCs was higher than their left counterparts. A similar higher VOR gain for rightward impulse direction has been reported [32-33]. This difference could be due to the limitation of the equipment used [32-35], or it could be due to the difference in head velocity between right and leftward movements [36-37]. In the case of the posterior canal, no right-left difference existed. 

Under the VNG test, caloric, saccade, smooth pursuit, and optokinetic tests were done. The caloric test was performed on 109 subjects, while others opted out. A bi-thermal air stimulation (50℃ and 24℃) was used in this study [38-40]. The test yielded symmetrical responses between the ears, which concurred with the previous studies [39-41]. Studies show that the air caloric test is equivalent to the water caloric test [40-42]. In the saccade test, the rightward and leftward peak velocities (for 10 and 15 degrees) of both eyes agreed with previous studies [43]. The present study's latency was less than 200 msec, and accuracy ranged between 85-95%. Some studies have reported slightly higher latency (>200 msec) [43-44]. Individual systems' sampling rates may induce this difference, and clinicians must know that [43]. In the smooth pursuit test, the rightward and leftward gain findings were similar to those reported in the literature [39, 43, 45]. The optokinetic task was performed with right and leftward moving stimuli at 20 degrees per second. There was no asymmetry between rightward and leftward movements. The findings were similar to those of previously published data [46,13]. Available normative data for vestibular tests are constrained by wide variability [11], the number of tests employed [12, 13, 15], test parameters reported [43,44], and sample size [11, 14]. Comprehensive normative data involving many test parameters on a largely homogeneous population could serve as a ready reckoner for clinicians. Studies such as this present research have tried to address the above issues. However, the participants of this study were from a wider range; further, the study does not provide normative data for narrow age ranges. Both of these issues are limitations of the present study.

## Conclusions

Normative data for vestibular tests are available; however, it is on limited tests and fewer samples. Comprehensive normative data involving all commonly employed vestibular tests in a large sample size would be helpful. This study brings forth normative data by evaluating 120 normal subjects between 18-55 years of age on various vestibular tests. The tests performed were cVEMP, oVEMP, vHIT, and VNG. The normative established by this study is in line with previously reported ones. The inverse relation observed between gain and stimulus frequency in the smooth pursuit test and the difference between horizontal and vertical gain needs further research. However, this data could be of help to both clinicians and researchers who work in the field of vestibular science. 
