# Correction: Comprehensive Normative Data for Objective Vestibular Tests

**DOI:** 10.7759/cureus.c132

**Published:** 2023-08-23

**Authors:** Suman Narayana Swamy, Pradeep Yuvaraj, Nupur Pruthi, Kandavel Thennarasu, Aravind Kumar Rajasekaran

**Affiliations:** 1 Speech Pathology and Audiology, National Institute of Mental Health and Neurosciences, Bangalore, IND; 2 Neurosurgery, National Institute of Mental Health and Neurosciences, Bangalore, IND; 3 Biostatistics, National Institute of Mental Health and Neurosciences, Bangalore, IND

This article has been corrected at the request of the authors to correct the pre-stimulus parameters in Table 1 from cVEMP = 20ms to 100ms and oVEMP = 20ms to 100ms.

In addition, the right and left ear maximum SPV and total SPV were corrected from

UW = RW + RC - LW + LC × 100 

RW + RC + LW + LC 

DP = RW + LW - RC + LC × 100 

RW + RC + LW + LC 

to 

UW = (RC-RW)-(LW-LC) / (RC-RW+LW-LC) × 100

DP = (RC+LW)+(RW+LC) / (RC-RW+LW-LC) × (-100)

The authors deeply regret that these errors were not identified and addressed prior to publication.

 

